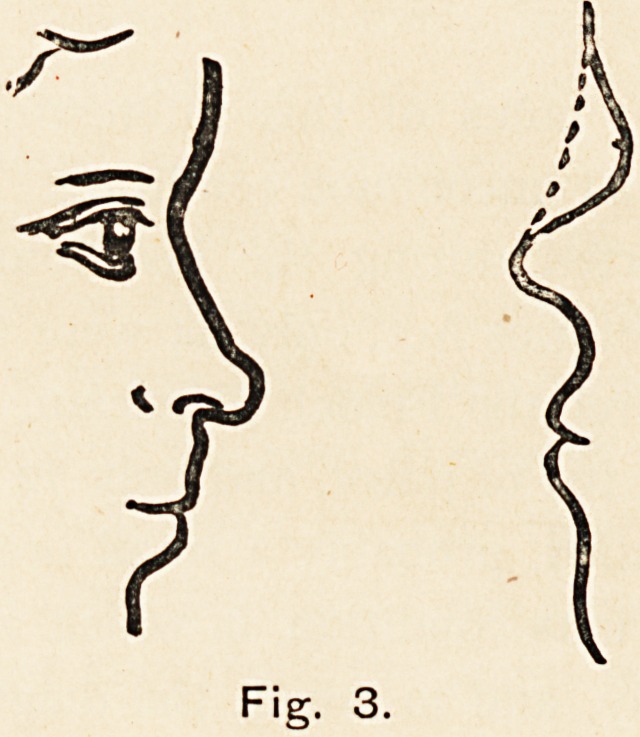# A Family with a Congenital Deformity of the Nose, and the Results of Subcutaneous Injection of Wax

**Published:** 1909-06

**Authors:** E. H. E. Stack

**Affiliations:** Surgeon to the Cossham and Orthopædic Hospitals; Assistant-Surgeon to the Bristol Royal Infirmary and Bristol Eye Dispensary


### A FAMILY WITH A CONGENITAL DEFORMITY OF THE NOSE, AND THE RESULTS OF SUBCUTANEOUS INJECTION OF WAX.

BY

E. H. E. Stack, M.B., B.C., F.R.C.S.,

*Surgeon to the Cossham and Orthopædic Hospitals ;*
*Assistant-Surgeon to the Bristol Royal Infirmary and Bristol Eye Dispensary.*

---

Since its introduction by Gersuny, there has been much written on the injection of paraffin for cosmetic and other purposes, and a good deal of discussion as to what melting-point at which the wax is best used. Many operators still advocate a wax which requires to be heated before it is injected. This is a troublesome method, as the wax cools so quickly when it runs down the needle and into the tissues ; it is also more difficult to limit its situation to the point required. Downie, who has done a large number of cases, uses an electric current to keep the needle hot. Others use quite a hard paraffin of the consistency of a candle, and without heating it, inject by considerable pressure. Three years ago I saw in the clinic of Gersuny in Vienna a large number of cases treated with wax, which had a melting-point of 35–40° C. At ordinary temperatures it is about the consistency of soft butter.

Gersuny now prefers this to harder or softer varieties. It does away with the trouble of keeping the wax hot while it is being put in on the one hand, and of having a special apparatus for the hard paraffin on the other. The technique is simple. The skin is cleaned, and the needle with a cocaine syringe is put in and a few drops injected. The syringe is taken off the needle, and the wax syringe (loaded) is screwed on instead. The wax is then gently forced into the tissues, which are held between the finger and the thumb of the hand. The amount injected depends on the result required. It is better to inject several times than run a risk of putting in too much at a time. When the skin become anæmic, no more should be injected at that time. The looser the skin is the better will be the result. No anæsthetic is required. I have never had a patient who found the pain more than they could bear quite easily ; in fact, I often do it without putting in any cocaine first. The needle can be moved from place to place as it is wanted till the proper contour of the part is reached. There seems to be no tendency for the wax to wander after the injection. It is now twenty-one months since the first of the noses I have shown was done. The patient has the same silhouette in profile now as then, and does not consider that any alteration has taken place.

I have injected several cases because of the difficulty they had in getting spectacles to sit on a depressed nose, and the results are satisfactory. They must, however, be careful not to have the bridge of the glasses too thin nor pressed too tightly against the skin. Many normal noses mark and even get sore with the pressure of the bridge of their glasses, and this is rather more apt to occur when there is wax, especially for the first few months. One patient (a cornet player) came to me on account of a depressed scar in his upper lip, the result of an old suppurating sebaceous cyst, which interfered with his proper lipping. I divided by subcutaneous puncture the fibrous band which bound down the skin, and a week later put in a tiny drop of wax with a very fair result, but I did not put in quite enough wax, and he is anxious to have a little more injected. The first case of a depressed scar I did was a patient who came to the Royal Infirmary for a strangulated hernia. He had a very ugly long scar on his chin, and seemed highly delighted when I offered to improve it for him, which I did. I heard afterwards that he was often " wanted " by the police, and so I am not surprised at his eagerness to have such a good identification mark removed. Scar cases should be done at two sittings. First dividing the cicatrix, and when this has healed injecting the wax. If the wax be put in at once it is liable to flow out through the hole made by the scalpel in releasing the scar. The effects are best watched if the needle be brought under the skin from some little distance. I have at present a case of small-pox pitting which I hope to show the Society next year. She is having first a course of massage to stretch the scars. The wax in these cases should be softened a little with some olive oil before injection. I believe the wasting of the socket after excision of an eye has been much improved by injection. In case anyone is interested in the future of the wax injected, I have put under the microscope a specimen of the wax before it is injected—it shows the usual needlelike crystals—and also a drop which I let out of a lip this afternoon six weeks after injection. The wax is there in very fine globules about the size mostly of red blood cells, some of which are seen in the field. Other cases which I have seen benefited are the cases of hemiatrophy of the face which Gersuny has published, flaccid elbow joint after excision, poor development of one side of the lower jaw, prolapse of the rectum ; but of these cases, although there have been some good results, there have also been several published where extensive suppuration has taken place along the wax planes, and I do not think I would be anxious to use it in such a case. For cases where too much wax has been put into a nose, or where it has been put in a wrong spot, Gersuny has a number of instruments which he uses to remove it, cutting only the mucous membrane ; with these one can also remodel a nose which the owner thinks is of too marked a Roman or Semitic type. There have been reported in Germany several cases where men have had themselves injected with wax in order to form tumours, and so avoid conscription. They have presented themselves at surgical clinics afterwards to have the masses removed from the scrotum, neck, &c., after it had served its purpose. One might, therefore, classify " the parafinomata " into unsurgical, unsightly and unpatriotic !

Turning now to the very interesting family of which I have shown you so many examples, I would like to have suggestions as to their pathology. They are all of the same type, though of a different degree. The eyes appear to be widely set, showing a tendency to epicanthus. The nose is extraordinarily broad, especially at its root. Fig. 1 shows a transverse section of one of these noses near the root, and of a normal one in the same situation. To describe its appearance, one might say that it looks as if the nasal process of the frontal bone had grown down between the nasal bones and separated then by a centimetre or more; this width is carried down to the tip, and there is no bridge. Just above the tip there is a pit or slight dimple, and in some cases this pit is almost the only evidence of the deformity. (*See* Fig. 3.) I have not been able to make out anything from skiagrams. Some few of the cases have chronic nasal catarrh, and

Fig. 1.

one has some ozœna from crusting. I have not been able to find references to any deformity of the sort; but I feel sure that such a marked condition must have been noticed before. Going into the family history, one finds, as shown on the genealogical chart, that the condition is known to be present in four generations. The youngest case, aged two years, is one of those you have seen downstairs, and is, perhaps, the most marked of all. Out of the forty persons in the tree, eighteen (viz. those surrounded by the

Fig. 2.

square) are the affected ones, nine are males and nine females; in most of them the deformity is well marked, but a few have only the dimple, although all show the broad nose. I have operated on nine out of the sixteen, and they are all very pleased with the result. I have not made up my mind yet with regard to the children as to when they ought to be done. I believe an operation to diminish the width of the nose would be successful, but I am at present a little loath to suggest it, as one could not be quite sure of the result. If I get a chance with a very young baby I will certainly try moulding. I may mention here that the deformity is quite noticeable at birth.

There is no female member of the family married (at present), and so one cannot say whether the deformity is more liable to pass through males or females. There is another curious feature about some of the cases, that is the deficiency of the hair in some of the children. They seem to grow out of it; but you see in the case to-night—who is two years old—she scarcely has any hair, and her elder sister is only just beginning to grow hers. I should be much obliged if anyone comes across a reference to a family

Fig. 3.

of this sort if they would let me know. The unaffected members of the family do not show any deformity, nor is there anything noteworthy about their appearance. The only suggestion I have to make about the pathology is that the suture between the two halves of the frontal bones closed late, and allowed the processes to be situated too widely apart. The mother of one family says that in some of the children there was a beating in the forehead just above the nose, and this would bear out the theory. It would have been interesting if one of the cases had had a frontal meningocele. The chart shows that the disease began with the youngest in a family of twelve. The sex of the other members of that family is not known, so I have put them alternately male and female, After this case there are four families. In two of these the percentage of abnormal to normal is 40, in one 100, and in the fourth 70. The case does not, therefore, fall into line with the dominants and recessives of Mendelism. The diagrams show (Fig. 3) the new line of the silhouette taken as a shadowgram before and after operation.

## Figures and Tables

**Fig. 1. f1:**
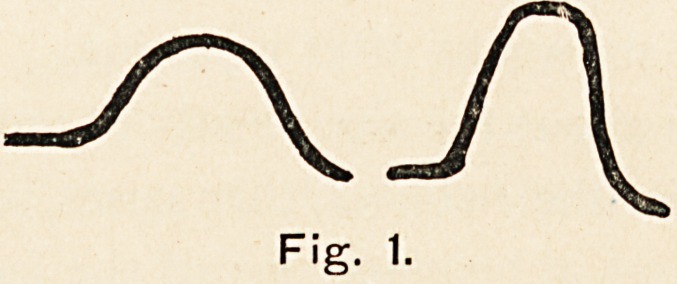


**Fig. 2. f2:**
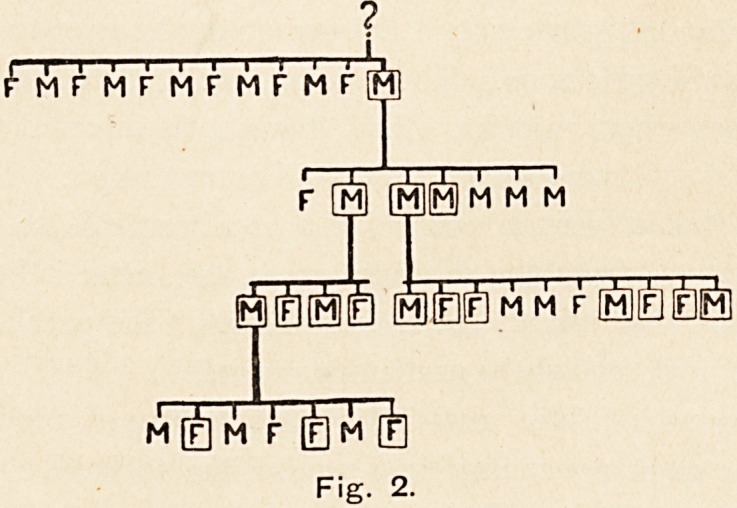


**Fig. 3. f3:**